# Artificial Intelligence-Enabled ECG Algorithm Based on Improved Residual Network for Wearable ECG

**DOI:** 10.3390/s21186043

**Published:** 2021-09-09

**Authors:** Hongqiang Li, Zhixuan An, Shasha Zuo, Wei Zhu, Zhen Zhang, Shanshan Zhang, Cheng Zhang, Wenchao Song, Quanhua Mao, Yuxin Mu, Enbang Li, Juan Daniel Prades García

**Affiliations:** 1 Tianjin Key Laboratory of Optoelectronic Detection Technology and Systems, School of Electrical and Electronic Engineering, Tiangong University, Tianjin 300387, China; 2030070833@tiangong.edu.cn (Z.A.); zhangshanshan@tiangong.edu.cn (S.Z.); zhangcheng@tiangong.edu.cn (C.Z.); 2031070927@tiangong.edu.cn (W.S.); 2030070834@tiangong.edu.cn (Q.M.); 2031070905@tiangong.edu.cn (Y.M.); 2 Textile Fiber Inspection Center, Tianjin Product Quality Inspection Technology Research Institute, Tianjin 300192, China; zuoshasha2021@gmail.com (S.Z.); zhuwei2120@gmail.com (W.Z.); 3 School of Computer Science and Technology, Tiangong University, Tianjin 300387, China; zhenzhang@tiangong.edu.cn; 4 Tianjin Key Laboratory of Optoelectronic Sensor and Sensing Network Technology, Institute of Modern Optics, Nankai University, Tianjin 300071, China; 5 Centre for Medical Radiation Physics, University of Wollongong, Wollongong, NSW 2522, Australia; enbang_li@uow.edu.au; 6 Institute of Nanoscience and Nanotechnology (IN2UB), Universitat de Barcelona (UB), E-08028 Barcelona, Spain; dprades@el.ub.edu

**Keywords:** biomedical monitoring, cloud computing, ECG science popularization, fabric electrodes, residual network

## Abstract

Heart disease is the leading cause of death for men and women globally. The residual network (ResNet) evolution of electrocardiogram (ECG) technology has contributed to our understanding of cardiac physiology. We propose an artificial intelligence-enabled ECG algorithm based on an improved ResNet for a wearable ECG. The system hardware consists of a wearable ECG with conductive fabric electrodes, a wireless ECG acquisition module, a mobile terminal App, and a cloud diagnostic platform. The algorithm adopted in this study is based on an improved ResNet for the rapid classification of different types of arrhythmia. First, we visualize ECG data and convert one-dimensional ECG signals into two-dimensional images using Gramian angular fields. Then, we improve the ResNet-50 network model, add multistage shortcut branches to the network, and optimize the residual block. The ReLu activation function is replaced by a scaled exponential linear units (SELUs) activation function to improve the expression ability of the model. Finally, the images are input into the improved ResNet network for classification. The average recognition rate of this classification algorithm against seven types of arrhythmia signals (atrial fibrillation, atrial premature beat, ventricular premature beat, normal beat, ventricular tachycardia, atrial tachycardia, and sinus bradycardia) is 98.3%.

## 1. Introduction

With the development of society, individuals are focusing more on maintaining their health. A growing number of people are relying on timely and effective technical approaches to safeguard their health and safety [1,2]. Although currently available medical monitoring devices exhibit certain advantages in terms of professionalism and accuracy, their monitoring time and scenarios are subject to certain restrictions. Furthermore, the ECG signals from the human body are relatively weak, low-frequency signals, which places a higher requirement for hardware acquisition equipment to obtain real-time and accurate ECG data. Consequently, effective long-term continuous monitoring cannot be achieved at any time and anywhere. With the popularization of smartphones, telemedicine service systems combined with smartphones are gradually emerging. The George Washington University in the United States developed a handheld electrocardiogram (ECG) recorder based on microelectronics. This recorder can record three-lead ECG data and display ECG in real time [3]. Although research on remote intelligent medical technology started late in China, relevant theoretical research is actively advancing. In 2019, Liu Chengyu et al. [4] of Southeast University developed a new type of wearable 12-lead ECG smart vest system based on the Internet of Things (IoT). This vest can be used for the early detection of heart diseases. Furthermore, the traditional ECG classification algorithm usually extracts and selects features manually, and this condition is not conducive to real-time detection. Lih et al. [5] proposed a system based on the combination of long and short-term memory (LSTM) and the convolution neural network (CNN) and obtained good results in the classification of five types of ECG signals. On the basis of all of the above-mentioned deep learning methods, ECG signals as 1D time series are input into a 1D-CNN. During this period, the gradient will disappear. Although LSTM alleviates this problem to a certain extent, it does not solve this problem completely.

The factors mentioned above gave us grounds to propose a wearable ECG monitoring and diagnosis system based on a cloud-computing platform. The system can continuously monitor the ECG activity of the body and transmit ECG data to a cloud-computing platform in real time. This system does not rely on a doctor to determine ECG status. In the cloud-computing platform, a set of heart rhythm recognition algorithms is designed on the basis of Python and MatLab (MathWorks, Portola Valley, CA, USA). This set of algorithms has high performance in terms of the precision and convergence of ECG signal classification and can discriminate and diagnose seven types of common arrhythmias in real time and send the diagnosis results to a mobile terminal App for display. Using this system, users can understand their heart conditions and receive timely and effective treatment, which is highly significant for the long-term monitoring of patients with chronic diseases. In addition, a display function for ECG science popularization is provided in this system, enabling users to gain information about ECGs by using the App. An in-depth understanding of cardiovascular and cerebrovascular disease prevention and health guidance could considerably improve the self-prevention level of patients.

## 2. Materials and Methods

**Figure 1:**
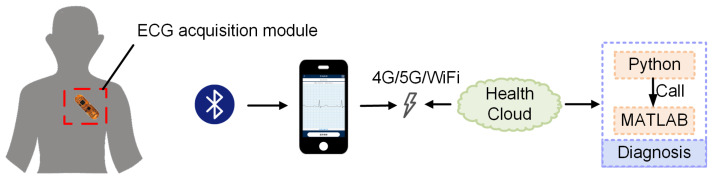
Schematic of overall system structure.

The wearable ECG monitoring and diagnosis system described in this study is primarily composed of wearable equipment, a mobile terminal App, and a cloud diagnostic platform. The overall structure of the system is shown in Figure 1. ECG signals are measured via a single-lead method in this design. The user’s ECG data are collected through the ECG acquisition module and processed by amplifying and filtering. Then, the processed data are sent to the mobile phone App through Bluetooth^®^ in real time. The App further processes the data and displays a dynamic ECG in real time. Meanwhile, the data are transmitted to the cloud diagnosis platform through a wireless network. The cloud diagnosis platform uses a heart rate recognition algorithm to classify and diagnose the data and then sends the diagnosis results to the App for display.

### 2.1. ECG Acquisition Module

**Figure 2:**
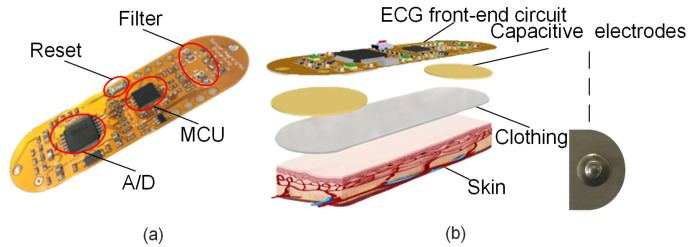
Signal acquisition module: (**a**) ECG signal acquisition circuit. (**b**) Exploded image of ECG acquisition module showing signal acquisition circuit, fabric electrodes, clothing, and skin.

ECG signals from the human body are relatively weak, low-frequency signals. Thus, designing an efficient hardware acquisition device is necessary to obtain real-time and accurate ECG data. The ECG signal acquisition circuit designed in this study is shown in Figure 2a. It is primarily composed of conductive fabric electrodes and an ECG acquisition circuit. In Figure 2b, the uppermost layer is the signal acquisition circuit, the sub-layer is the fabric electrode sheet, the third layer is the clothing, and the fourth layer is the user’s skin.

#### 2.1.1. Conductive Fabric Electrodes

Traditional electrodes are irritating and cannot be reused. Thus, conductive fabric electrodes measuring 2.5 cm × 1.8 cm were designed and fabricated under the capacitive coupling principle, as shown in Figure 2b. The conductive fabric is laid over polyester fiber and then plated with nickel. Then, a high-conductivity copper layer is placed on the surface of the nickel. The copper layer is again plated with anti-oxidation and anti-corrosion nickel. The conductive fabric produced in this manner can provide excellent conductivity. The conductive fabric electrode is extremely flexible because its thickness is only approximately 0.11 mm. Thus, it is suitable for human skin, providing an effective method for achieving long-term continuous real-time monitoring. Moreover, preparation operations are not required for skin monitoring if the conductive fabric electrode is used. No symptoms, such as those due to allergies, will manifest on human skin. This electrode can be implanted into clothes, considerably improving patient comfort.

#### 2.1.2. ECG Acquisition Circuit

**Figure 3:**
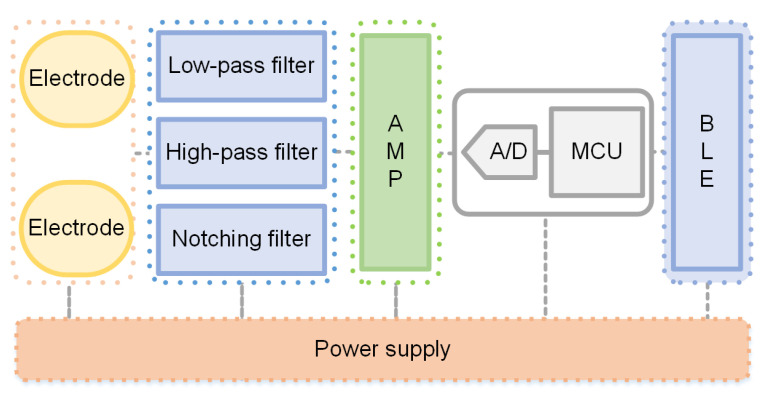
Block diagram of signal acquisition circuit.

In this study, ECG signals were measured via a single-lead method. ECG signals are relatively weak, and thus they are susceptible to electromagnetic waves and other interference factors during the signal acquisition process. Accordingly, an ECG signal acquisition circuit is required to filter and amplify ECG signals. The signal acquisition circuit described in this paper has five modules: signal acquisition, signal filtering and amplification, signal processing, power supply, and Bluetooth^®^. The principal block diagram of this circuit is provided in Figure 3.

The ECG acquisition circuit uses ADI’s ADuCM361 chip as its primary control chip. The ADuCM361 chip exhibits the advantages of low power consumption, high real-time performance, rich on-chip peripherals, and large storage space. The second-order Butterworth low-pass filter of the voltage-controlled voltage source is used as the low-pass filter of the circuit, and the cutoff frequency is 1.5 Hz. Meanwhile, a voltage-controlled voltage source second-order Butterworth high-pass filter is used as the high-pass filter of the circuit. A notch filter is designed with a symmetrical double-T active filter network with a cutoff frequency of 50 Hz.

The amplitude of ECG signals is extremely small, and thus amplifying ECG signals is necessary. Moreover, given that the collected ECG signals are analog signals, analog-to-digital conversion (ADC) is necessary. The ECG signal acquisition circuit uses ADI’s low-power, five-channel ECG signal-dedicated ADAS1000 chip for ADC. This chip can simultaneously perform signal gain and ADC, simplifying the design of the ECG acquisition circuit.

After completing ECG data collection and signal processing operations, the ECG signals are sent to the mobile phone client via Bluetooth^®^, and then the App shows a real-time ECG waveform diagram and saves the data; at the same time, the App receives ECG data uploaded to the cloud, which uses Python to receive and process data, where the processing relies on the MatLab rhythm of diagnosis. Finally, the diagnostic report is fed back to the App for users to view. To ensure that the ECG acquisition system has sufficient battery life, Bluetooth^®^ Low Energy (BLE) is used as the communication bridge between the wearable devices and mobile phone clients. Moreover, a light-emitting diode status indicator is added to the Bluetooth^®^ module. When the App is successfully paired, the indicator always remains on, and ECG data can be collected and transmitted in real time.

### 2.2. ECG Classification Algorithm

We propose an ECG signal classification method based on the combination of Gramian angular fields (GAFs) and an improved deep residual network (ResNet) to provide an accurate and rapid diagnosis for the sensors. In this work, a one-dimensional (1D) ECG signal was converted into polar coordinates using this algorithm based on the data visualization method of GAFs. Then, the coordinates were encoded to a 2D image using angle information. Thereafter, an improved ResNet structure was built, and five groups of shortcut connections were added into the original ResNet-50 model to adjust the structure of the residual block. The downsampling operation in the trunk path of the residual block was placed in the convolution layer with the size of 3 × 3. Batch normalization (BN) and activation operations in the bottleneck block in the residual structure were pre-positioned. The average pool layer was added to the bypass of the residual block. A SELU activation function was also used to replace the ReLu activation function. Finally, a good result was achieved by testing seven kinds of ECG data extracted from the MIT-BIH arrhythmia database, long-term AF (LTAF) database, and Prosim2 vital sign simulator.

Deep learning can extract effective features from the original data and output the classification results. It has achieved good results in the fields of computer vision and speech recognition [6]. The data processing method of GAFs [7] was adopted to encode a 1D ECG signal into a 2D image in the present work to fully utilize the advantages of deep learning in image classification.

**Figure 4:**
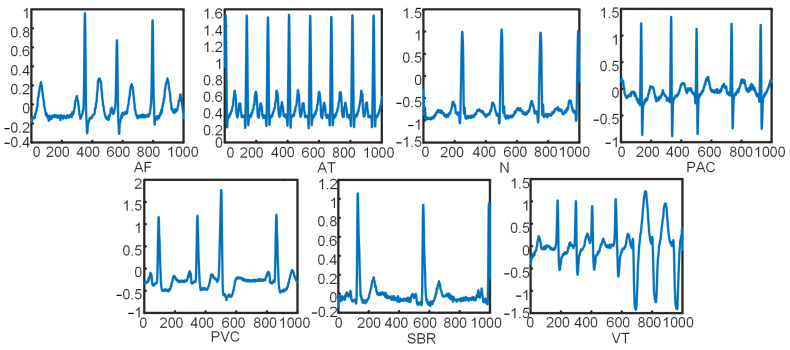
Seven types of original ECG data.

**Figure 5:**
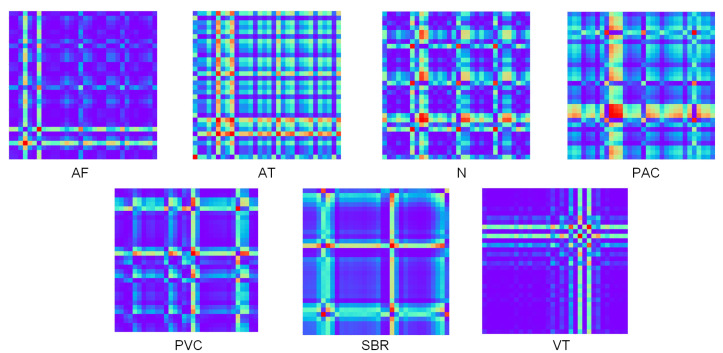
GASF images.

Figure 4 shows the seven types of original ECG data that were extracted. Gramian Summation Angular Field (GASF) images converted through GAF data processing are shown in Figure 5. Compared with original data, GASF has two advantages. First, GASF provides a way to maintain time dependence. Time increases as the position moves from the upper left-hand corner to the lower right-hand corner. Second, GASF contains time correlation. The diagonal is composed of the original values of the normalized time series. The main diagonal can be used to reconstruct time series from the advanced features of deep neural network learning.

#### 2.2.1. Deep ResNet and Its Improvement

**Figure 6:**
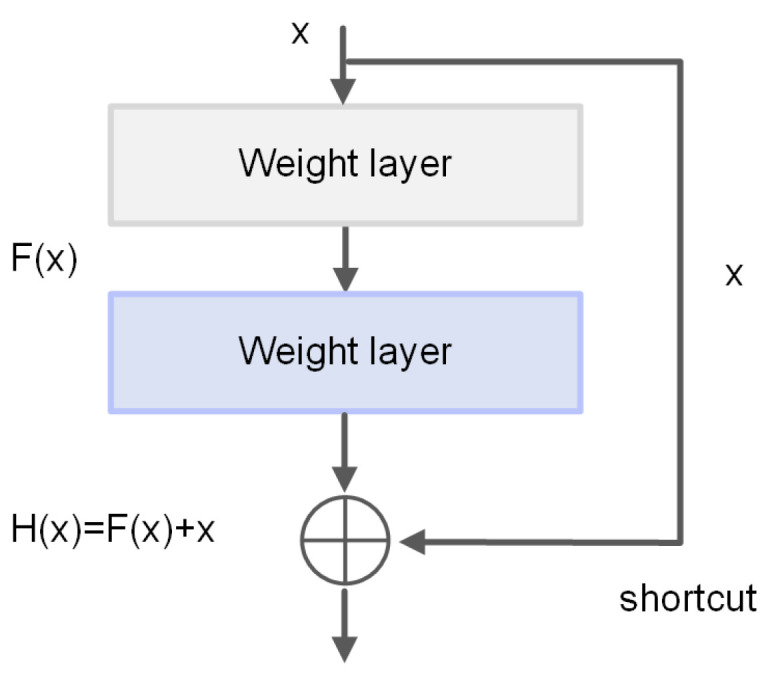
Residual structure.

K. He [8] proposed ResNet. This network can directly transfer the current output to the next layer of the network by adding identity mapping. No additional parameters are added due to the one-to-one transmission. At the same time, the gradient of the next-layer network is directly transferred to the upper-layer network in the process of back-propagation. This approach solves the problem that the deep neural network degrades with the deepening of network convolution layers. A BN algorithm is used to accelerate the convergence of the network and improve training speed. This ResNet structure can expand the network to more than 1000 layers, and the final classification effect is also good. The structure of the residual block is shown in Figure 6. If the output is set to x and the expected relation is mapped as H(x), then the output result is H(x)=F(x)+x. If F(x)=0, then H(x)=x; that is, so-called identity mapping. ResNet changes the learning objectives. F(x) is used as the target value; that is, the so-called residual F(x)=H(x)−x. As a result, the gradient transmission of identity transformation and back-propagation can be ensured, which alleviates the network degradation.

**Figure 7:**
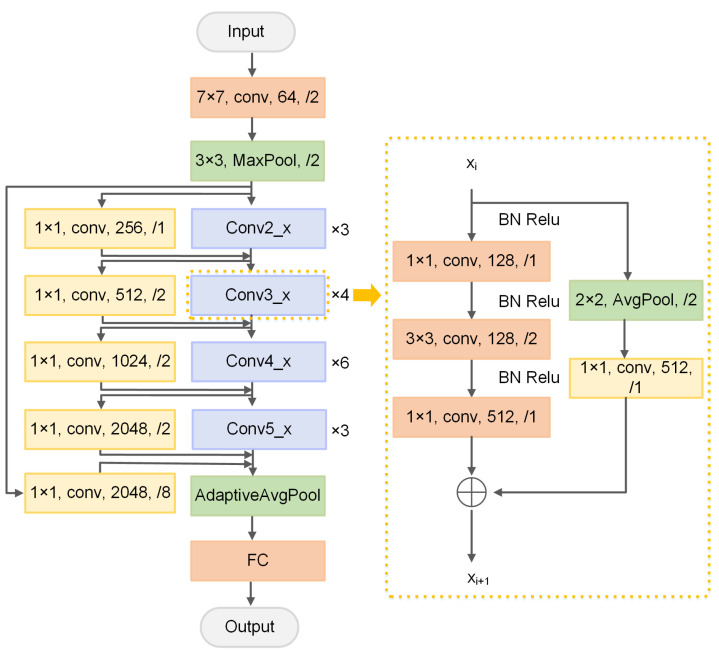
Improved ResNet structure.

In the proposed improved scheme, multi-layer shortcut branches are added [9] on the basis of ResNet-50 to improve the structure of the residual block. The structural framework is shown in Figure 7. After passing through 7 × 7 convolution and pooling layers, this model passes through four groups of residual blocks composed of Conv2_x, Conv3_x, Conv4_x, and Conv5_x. The four groups of residual blocks contain three, four, six, and three residual blocks, respectively.

The specific structure of the residual block is shown in the right-hand part of Figure 7. This structure changes the downsampling block of the main path of the residual block. The step length in the original 1 × 1 convolution layer is set to 1 from 2, and the step length in the 3 × 3 convolution layer is set from 1 to 2. The convolution kernel of the first convolution layer in the original residual block is 1 × 1. A step length of 2 will lead to three-quarters of information loss after convolution. Therefore, the downsampling operation is applied to the 3 × 3 convolution layer. Similarly, the step length of the 1 × 1 convolution layer in the bypass of the residual block is also set to 1. A 2 × 2 average pooling layer is added in the bypass 1 × 1 convolution layer with a step length of 2 to keep the output dimension of the bypass and trunk path consistent. The input dimension of Conv2_x is the same as the output dimension. Thus, the average pooling layer is not added in the bypass of the Conv2_x residual block, but the average pooling layer is added in only the bypass of the first residual block of Conv3_x, Conv4_x, and Conv5_x. Inspired by [10], the BN layer in the trunk path of the residual block and activation operation are pre-positioned. This approach eases the back-propagation and optimization of the gradient. The use of BN in pre-activation can also improve the regularization of the model.

Multi-layer shortcut branches are added in each group of the residual block in the entire network. Specifically, a layer of shortcut connections is added in addition to three residual blocks of Conv2_x. The step length is set to 1 to ensure consistency with the output dimension of the trunk path of the network. A layer of shortcut connections is also added in Conv3_x, Conv4_x, and Conv5_x with a step length of 2. A layer of shortcut connections is also added outside the entire residual block with a step length of 8. The size of the convolution kernel in the newly added multi-layer shortcut branches is 1 × 1. Learning objectives can be transformed into learning residual-to-residual mapping by adding multi-layer shortcut branches. This mapping is simpler and easier to learn than the original network. It can also spread information in different residual blocks. This approach can alleviate the problem that the gradient disappears to a certain extent and facilitate the better training and classification of the network.

#### 2.2.2. SELU Activation Function

In most CNNs, ReLu is used as the activation function. However, the gradient of ReLu is 0 in x<0, and this function easily causes neuron death. A ReLu activation function is defined as follows:

(1) $$f\left(x\right)=\left\{\begin{array}{cc} x, & \left(x\geq 0\right)\\ 0, & \left(x<0\right) \end{array}\right.$$

**Figure 8:**
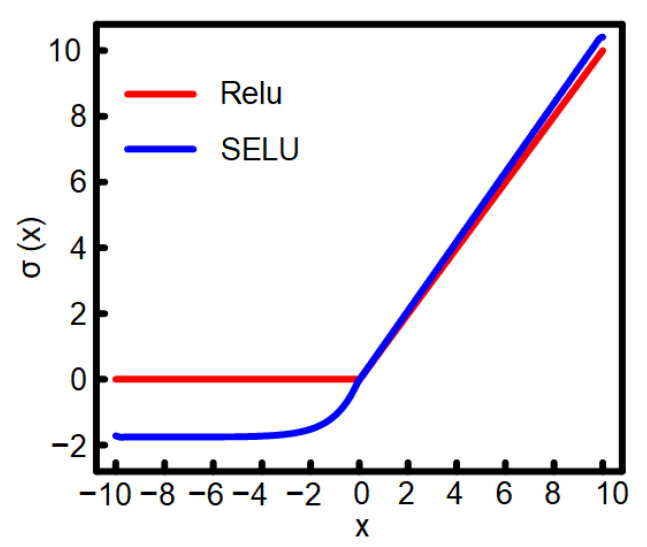
ReLu and SELU activation functions.

Figure 8 shows that, when x>0, the derivative of ReLu is always 1, which can hinder the attenuation of the gradient at x>0. As a result, the problem that the gradient disappears is solved. However, when x<0, the gradient of ReLu is 0. During the period of training, the negative gradient is set to zero on ReLu, which results in the corresponding neurons never being activated. As a result, the corresponding weight cannot be updated; that is, neuron death occurs.

The convergence rate of the SELU activation function is faster than that of the ReLu activation function. After the activation function, the sample distribution is automatically normalized to 0 mean and unit variance to ensure that the gradient will not explode or disappear in the training process [11]. The SELU activation function is defined as follows:

(2) $$SELU\left(x\right)=\lambda \left\{\begin{array}{cc} x, & \left(x\geq 0\right)\\ \alpha e^{x}-\alpha , & \left(x<0\right) \end{array}\right.$$

where α≈1.6733 and λ≈1.0507. The SELU activation function is gentle on the negative half-axis. In this way, it can be reduced when the variance is too large in the activation operation to prevent gradient explosion. In contrast, the slope of the positive half-axis in ReLu is λ, which is a number greater than 1. It can be increased when the variance is too small to prevent the disappearance of the gradient at the same time. As a result, the activation function produces a fixed point. Even if the network deepens, the output of each layer is 0 mean and 1 variance. Therefore, the SELU activation function is used to replace the ReLu activation function to optimize network training.

In this study, the PyTorch deep learning framework was adopted to construct the network. Python was adopted for programming. The operating environment was Windows 7, with 32 GB of memory, an Intel E5-2620 processor, and an NVIDIA Geforce GTX 1080Ti graphics card.

#### 2.2.3. Source of Dataset and Evaluation Index

The data in this experiment used to verify the effectiveness of the constructed model were taken from the aforementioned MIT-BIH arrhythmia database, LTAF database, and Prosim2 vital sign simulator. Three types of ECG—namely, premature atrial contraction (PAC), premature ventricular contraction (PVC), and normal (N)—were extracted from the MIT-BIH arrhythmia database. Three types of ECG—namely, atrial fibrillation (AF), ventricular tachycardia (VT), and sinus bradycardia (SBR)—were extracted from the LTAF database. Atrial tachycardia (AT) was extracted from the Prosim2 vital sign simulator. A total of 11,724 groups of data were extracted. The training and testing sets were randomly allocated in the ratio of 8:2. The distribution of experimental sample data is shown in Table 1.

The precision rate (*Ppr*), sensitivity (*Sen*), specificity (*Spe*), *F*1 score, and recognition accuracy (*Acc*) are used as evaluation indexes in this work to facilitate the performance evaluation of ECG signal classification. The calculations are as follows:

(3) $$Ppr=\frac{TP}{TP+FP}$$

(4) $$Sen=\frac{TP}{TP+FN}$$

(5) $$Spe=\frac{TN}{TN+FP}$$

(6) $$F1=\frac{2\times Ppr\times Sen}{Ppr+Sen}$$

(7) $$Acc=\frac{TP+TN}{TN+FP+TP+FN}$$

where *TP* refers to true positive, which represents the number of samples corresponding to the correct classification of Y-type ECG signals; *TN* refers to true negative, which represents the number of samples corresponding to the correct classification of non Y-type ECG signals; *FP* refers to false positive, which represents the number of samples corresponding to the false classification of other types of ECG signals into Y-type ECG signals; *FN* refers to false negative, which represents the number of samples corresponding to the false classification of Y-type ECG signals into other types of ECG signals. The precision rate represents the proportion of correct predictions of Y-type ECG signals to all predictions of Y-type ECG signals; sensitivity represents the proportion of correct predictions of Y-type ECG signals to all Y-type ECG signals; specificity represents the proportion of correct predictions of non Y-type ECG signals to all non Y-type ECG signals; the accuracy rate represents the proportion of all correctly predicted samples to total samples; and the *F*1 score represents the weighted average of the precision rate and sensitivity, for which the best value is 1 and the worst 0. The *F*1 score was introduced as a comprehensive index to balance the influence of precision rate and recall. It can reflect the overall classification ability of the system on the whole. If its value is greater, the classification result is better.

#### 2.2.4. Comparative Network Experiment

**Figure 9:**
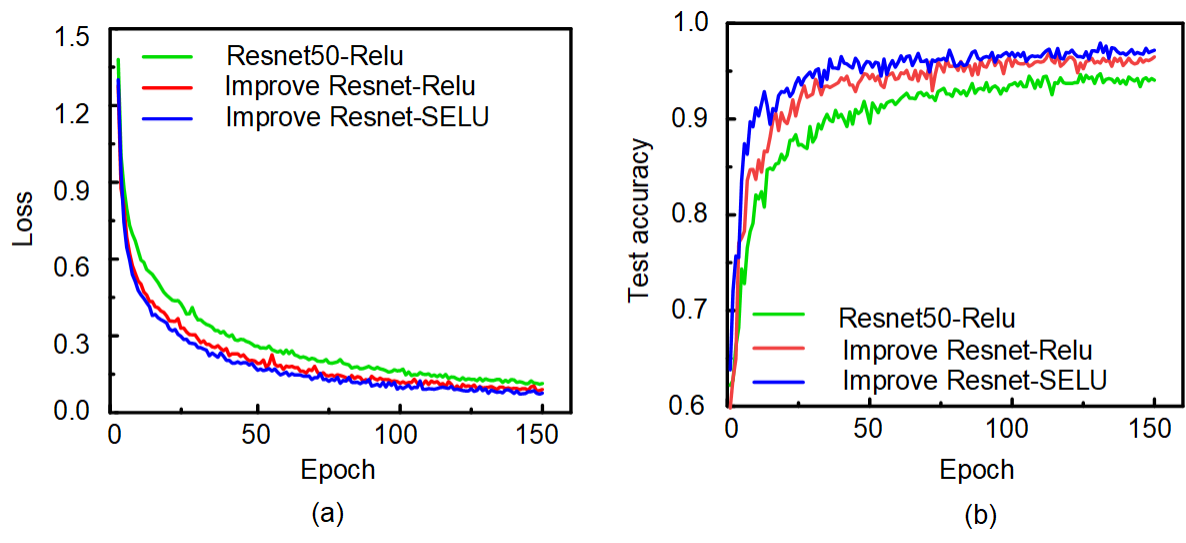
Training results: (**a**) Loss curve. (**b**) Accuracy curve.

In this experiment, the original ResNet-50 model, improved ResNet-ReLu model, and improved ResNet-SELU model were used to test the ECG data. Adam was selected as an optimizer. The initial learning rate was set to 0.0001 and the batch size to 32. In other words, 32 images and corresponding tags were selected from the input images each time as a batch for network training. A total of 150 epochs were trained in the network. The training result is shown in Figure 9. With the increase of iterations, the loss value of the network dropped rapidly and tended to be stable, and the loss value of the improved ResNet network was smaller than that of the original ResNet network. In the training process, the test accuracy rate of the network increasesd rapidly with an increasing number of iterations and gradually tended to be stable. The recognition accuracy rate of the improved ResNet network was higher than that of the original network. Moreover, the classification effect of the SELU activation function was better than that of the ReLu activation function (as shown in Table 2). Notably, the ECG signal recognition system based on the improved ResNet showed good performance.

#### 2.2.5. Confusion Matrix and Indicators

The classification confusion matrix of seven types of ECG signals produced by the algorithm proposed in this study is shown in Table 3. Each row of the matrix represents the real category of data, each column represents the predicted category, the total data in each row represent the actual number of test data of this category, and the numerical value of each column represents the number of real data predicted as this category. The precision rate, sensitivity, specificity, accuracy rate, and F1 score can be calculated based on the confusion matrix. The calculation results are shown in Table 4. As observed, the precision rate, sensitivity, specificity, F1 score, and recognition accuracy rate obtained by the proposed algorithm were all greater than 97%. This result shows that the algorithm has high stability and accuracy.

### 2.3. Mobile Terminal App

**Figure 10:**
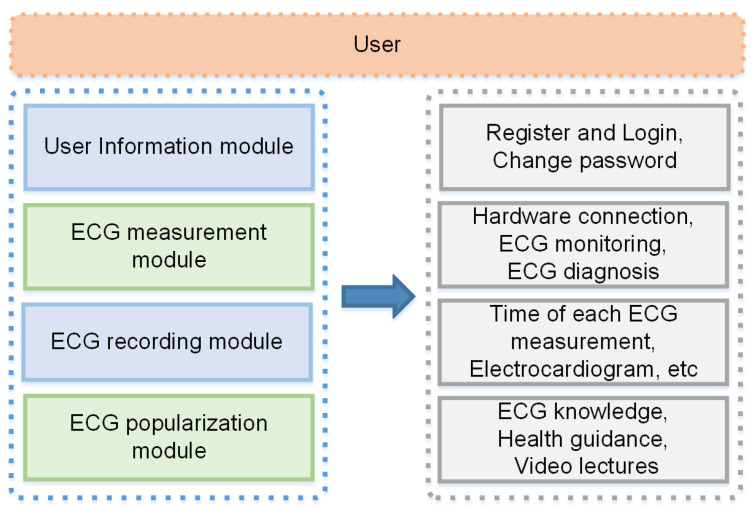
Block diagram of App function module.

To improve users’ understanding of their ECG activity, a mobile App based on the Android OS was developed in this study. The major functions of the App can be divided into four parts as follows (Figure 10):

1. User information management;
2. ECG signal receiving and display;
3. Historical measurement information recording;
4. ECG science knowledge display functions.

After the user registers and logs in, Bluetooth^®^ pairing is first performed, and the ECG data sent by the ECG acquisition module are further processed. Then, the ECG waveform diagram of the processed data is drawn, and finally the ECG data are transmitted to the cloud diagnosis platform for diagnosis and the diagnosis results are sent to the App. A display function for ECG science popularization has been added to this App, allowing users to gain scientific knowledge by using it.

#### 2.3.1. User Registration and Login

To protect the safety of the user’s personal information and provide personalized health monitoring services, a user information management module was designed. The module is divided into three functions: user registration, login, and password modification. The RxJava + Retrofit2 + OkHttp3 framework is used in the network request function involved in this process. The following components of the framework have their respective responsibilities: Retrofit uses an interface method to define the requested URL and return the value type; OkHttp is responsible for the request process, such as the GET and POST request methods; and RxJava is responsible for asynchronous operations and switching between threads. The combination of the three components makes network requests in Android development simple and well-designed. The user’s basic information is saved in a MySQL database. Finally, the user’s login password is encrypted with MD5 to improve user information security.

#### 2.3.2. Reception and Display of ECG Signals

ECG measurement is one of the most important functions of the App, which requires the realization of the real-time monitoring of the user’s heart condition. During measurement, the user must first use the BLE communication function to connect the App to the front-end ECG acquisition device. After clicking “Receive Data”, Bluetooth^®^ begins to receive ECG data, and the dynamic ECG waveform is displayed in real time on the mobile phone. Given that the application window is not required to be redrawn when the SurfaceView window is refreshed, the key technologies adopted for realizing the Holter waveform are Android SurfaceView and Canvas. After receiving data for a certain period, “Stop Receiving” is clicked. At this time, the ECG data and Holter waveform are saved in the database, and the ECG data are sent to the cloud diagnostic platform for heart rhythm recognition and diagnosis through the network via socket communication technology. Finally, the cloud diagnostic platform sends the diagnosis results to the App.

#### 2.3.3. Record of Historical Measurement Information

To enable users to understand their recent ECG activity, each bit of ECG information measured by the App is stored in a SQLite database and displayed to users in a list. An open-source framework—i.e., GreenDao—is used in the App to manage the SQLite database. The adoption of GreenDao enables the database to operate more quickly and saves development time. In this module, users can choose to retain or delete each record in accordance with their needs. They can also browse the Holter and diagnosis records again when necessary.

#### 2.3.4. Popularization of ECG Knowledge

**Figure 11:**
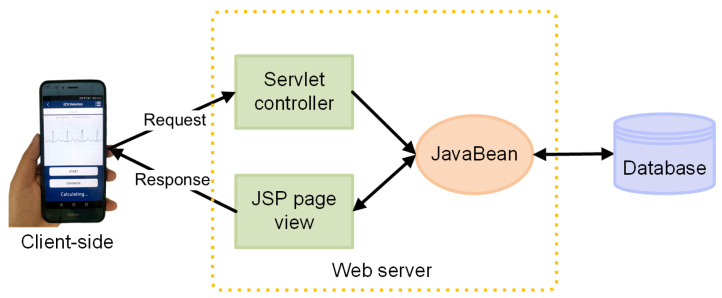
JSP, JavaBean, and Servlet model.

To strengthen the user’s personal monitoring, a method of displaying popular ECG science knowledge is introduced in this work. The main technologies used for this include the Tomcat container and Servlet technology, using Tencent Cloud as the server and finally realizing the network communication between the mobile App and server. Web and Android hybrid development technology is used for this part, and WebView control is added to the Android layout to load HTML5 pages for display (Figure 11). Web development primarily uses JSP as the view, the Servlet as the controller, and JavaBeans as the model. JSP enables the interaction with the App. The Servlet is the communication bridge between JSP and JavaBeans, and it is used for data interaction with the App. JavaBeans realizes the business logic of the system; i.e., it encapsulates the attributes of objects and connects to the database.

The specific steps of the ECG popular science module receiving a URL and retrieving data from the database for display are as follows. Servlets are used to handle HTTP requests. First, parameters (categories of popular science knowledge) are obtained on request:

String category = request.getParameter(“category”)

All popular science knowledge items of this category in the database are obtained by the specified category and saved in a set:

List<Knowledge> knowledgeList = service.getByCate(“category”)

Then, the collection is put into the request domain and forwarded to the page for display. The page takes out the collection in the request domain and traverses it through JSTL and EL expressions, finally showing the popular science content to users.

The click event of each popular science message is the same, but the parameters are different. Thus, different popular science information corresponds to a different HTML5 interface. First, JavaScript is used in the background to set the click event of the popular science button, and then a JavaScript interface is added to the WebView on the mobile terminal to implement the toDetail() method. After calling this method, the page will jump to Guid1Activity, and the parameter “id” is received on this page to determine the details of the popular science information corresponding to each topic.

**Figure 12:**
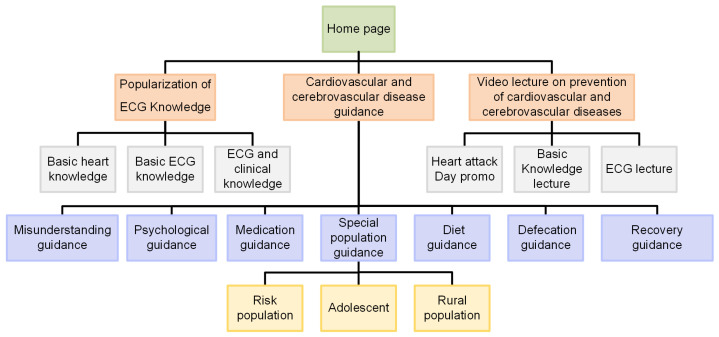
System content structure diagram.

As shown in Figure 12, the popular science knowledge module first displays basic heart knowledge, basic ECG knowledge, and ECG and clinical knowledge. Second, it provides guidance on prevention, misunderstanding, psychological, medication, special population, diet, defecation, recovery, exercise, and other cardiovascular and cerebrovascular disease. Finally, it displays heart-related video lectures, such as promotional videos, basic heart knowledge, and basic ECG knowledge.

### 2.4. Implementation of Cloud Diagnostic Platform

The ECG data of the mobile phone client are displayed through the ECG acquisition module. It is necessary for the collected ECG signals to be analyzed and diagnosed; then, the diagnosis results are sent back to the App. The results require more complex calculation methods, such as data pre-processing, feature extraction, and classification diagnosis. However, the mobile phone client App is only suitable for simple data processing, and it cannot realize the aforementioned complicated calculations. Therefore, the algorithm for ECG diagnosis is transferred to the cloud, and the environment and code required to execute the algorithm are built in the cloud server. The monitoring system designed in this study was based on Tencent Cloud’s Windows Server 2012. Although Python has been popular in recent years and exhibits good scientific computing capabilities, a Python/MatLab mixed programming method is used to calculate and diagnose ECG data because MatLab is a conventional and powerful mathematical simulation software with machine-learning libraries and other artificial intelligence algorithm resources. However, MatLab cannot perform network communication. In this approach, Python is responsible for data communication between the network interface and the App. Meanwhile, MatLab is used to implement the ECG classification method. The Python terminal calls MatLab functions or scripts after receiving ECG data. Thereafter, the ECG classification method based on the improved ResNet in MatLab is used to perform the pre-processing, feature extraction, and classification diagnosis of ECG data.The hit rate of the proposed algorithm is 98.3%. Finally, the diagnosis results are sent to the App.

#### 2.4.1. Python Internet Modules

The Python Internet module is implemented through the Python network socket library, which is typically included in other major programming languages, such as C++ and Java. Socket network programming has several major functions, such as bind(), listen(), accept(), send(), and receive(). Python is relatively flexible and easy to study. It has been widely used in recent years and has become popular. For the system developed in this work, the Python socket module provides an interface for receiving ECG data from the mobile terminal App and returning calculation results to the App.

#### 2.4.2. Python/MatLab Mixed Programming

Python/MatLab mixed programming is used to process and analyze the collected ECG signals. Python is responsible for data communication with mobile terminals, while MatLab is responsible for data calculation and diagnosis. Python and MatLab can be connected and called through the official driver “matlab.engine”. Thus, the built-in functions of MatLab can be directly called in their original form in the Python environment. The specific achieving logic is as follows.

**Figure 13:**
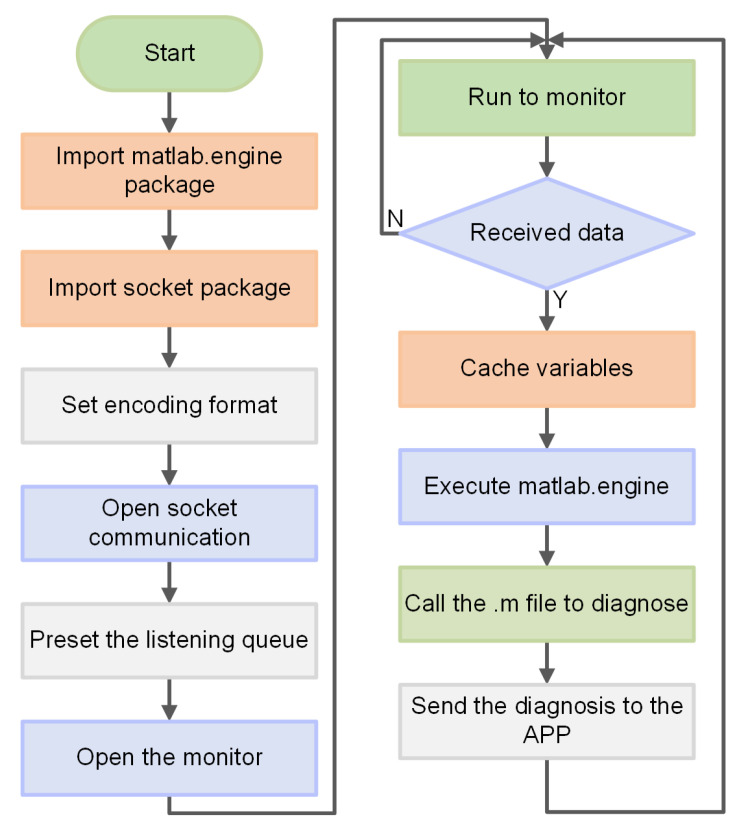
Specific process of the cloud server’s signal processing.

First, the corresponding IP address and port number are set through the socket to receive the data sent by the mobile terminal, and the received data are saved as data.txt. Then, every 2000 pieces of data are processed separately, and the SVMPSO3 ECG classification approach in MatLab is called by executing the ret = matlab.engine.start_matlab(). SVMPSO3(matlab.double()) code is used to classify and diagnose the data and assign the result to ret. Finally, the processed data diagnosis result is sent to the mobile terminal through socket.send(). The specific ECG signal processing and diagnosis flowchart is shown in Figure 13.

### 2.5. Experimental Tests

The ECG signals of patients were collected by wearable ECG devices to verify the practicability of the algorithm. The data were sent to the mobile App through Bluetooth^®^, and the data were then sent to the server by the mobile App. The proposed algorithm was adopted to test ECG signals in the server.

**Figure 14:**
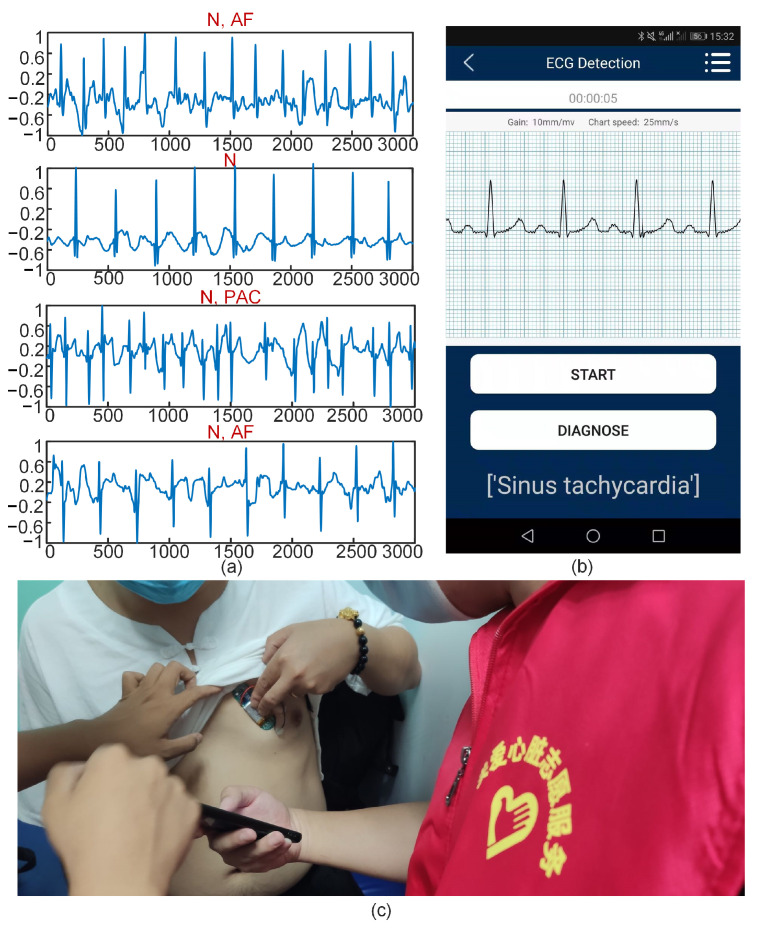
Clinical data collection and the deep neural network results: (**a**) Collected ECGs of four patients, (**b**) App real-time monitoring and diagnosis, (**c**) Clinical test of hospital patients.

To verify the practicability of the developed ECG monitoring and diagnosis system, the research team organized personnel to visit the outpatient department of the Department of Cardiology of Tianjin Chest Hospital to provide free consultations to patients (Figure 14). The collected ECGs of four patients are shown in Figure 14a. Table 5 shows the diagnosis results. Patient No. 1 was a patient with AF. The results of algorithmic diagnosis showed two types of ECG signals—namely N and AF—in the patient’s ECG signal. Patient No. 2 was a patient with incidental PAC. The patient did not have symptoms of PAC in the examination, and the test result was N. Patient No. 3 was a patient with frequent PAC. The test results showed N and PAC in this patient’s ECG signals. Patient No. 4 was a patient with PVC and AF. On the day of the examination, the patient took drugs, and thus no symptom of PVC was observed. The result showed two types of ECG signals: N and AF.

In summary, the diagnosis result using the algorithm was consistent with the doctor’s diagnosis result. This consistency further verifies the good performance of the proposed algorithm for ECG signal classification.

## 3. Discussion

An artificial intelligence-enabled ECG algorithm based on improved ResNet for a wearable ECG device is proposed in this paper. First, a GAF is adopted to convert 1D ECG signals into 2D images, which retains time dependence and strengthens the advantages of deep learning in ECG signal classification. Second, the ResNet-50 network is improved. Multi-layer shortcut branches are added to improve the structure of the residual block. At the same time, a SELU activation function is used to replace the ReLu activation function in the original network, which improves the nonlinear expression of the network and inhibits the death of neurons to a certain extent. In the experiment, 12,447 groups of ECG data were extracted from the database to train and test the network. The accuracy rate reached 98.3%.

In terms of the accuracy rate, the comparison of the ECG signal classification results between the proposed method and those in other studies is shown in Table 6. In [12], an ECG signal classification algorithm based on a spiking neural network (SNN) is proposed. The learning rules of Spike Timing Dependent Plasticity (STDP) and the inhibition layer are optimized, and the accuracy rate is 97.9% and the F1 score is 88.0%. Compared with the accuracy rate and F1 score of this method, the results of the proposed method are improved by 0.4% and 9.1%, respectively. In [13], an ECG signal-classification-algorithm-based multi-layer perception (MLP) is proposed. Compared with the accuracy rate and F1 score of this method, the results of the proposed method are improved by 3.5% and 19.8%, respectively. In [14], a wavelet transform is adopted for feature extraction first; then, principal component analysis is used for dimension reduction; finally, a MLP optimized by particle swarm optimization is used for classification. By recognizing five types of ECG signals, the accuracy rate and F1 score obtained are 95.4% and 96.8%, respectively. In [15], a classification algorithm based on the combination of a 16-layer CNN and short-term memory network is proposed. The accuracy rate achieved by the proposed algorithm is increased by 2.9% and the F1 score is increased by 0.3% compared with this method. The results show that the proposed algorithm has a high accuracy rate and a certain clinical application value. In terms of the computational power, in [16], a multi-sequential approach with four stages is proposed to discriminate among various malignant arrhythmias, with an accuracy rate of more than 95% for VF, AF, and PVC. In [17], a novel density Poincare plot-based machine learning method to detect AF from PAC/PVCs using ECG recordings is proposed, and the accuracy rate of AF detection from PAC/PVCs recorded by ECG was 97.45%. In Ref. [18], a robust algorithm is proposed for automatic detection of AF based on the randomness, variability, and complexity of heart beat interval time series, with an accuracy rate of 90.2%. Although the performance of these three methods is almost as good as that of the method in this paper, the computational power is significantly lower. The method based on an improved ResNet proposed in this paper can directly identify seven types of ECG signals of AF, AT, N, PAC, PVC, SBR, and VT with higher computational power.

Although the research content in this paper has achieved good results in both a simulation experiment and actual human body detection, there are still some study limitations. Firstly, the algorithm adopts a convolutional neural network, which needs to convert the input data into images. Using too many samples will occupy a great deal of memory space. Secondly, if popular science application software for ECG monitoring wants to realize the diagnosis of ECG signals, it must be realized by calling the MatLab algorithm with a Python background, which may be limited by the network. Therefore, we plan to embed ECG diagnosis algorithm into the App in the future. In this way, intra-App ECG signal diagnosis can be realized across network constraints.

## 4. Conclusions

The ECG monitoring approach detailed in this study achieved the collection and management of user ECG signals; particularly, the long-term and real-time collection of ECG signals. Moreover, the ECG data in the system were analyzed and diagnosed, and the corresponding diagnosis results obtained using an ECG de-noising method based on an improved ResNet. The accuracy rate reached 98.3%. In addition, the actual tests were conducted in a hospital. The results are consistent with the type of arrhythmia diagnosed by doctors and show that the proposed network model has high performance in terms of the precision and convergence of ECG signal classification. The proposed method also performs better than the traditional deep ResNet, and this performance proves its effectiveness and practicability.

## Figures and Tables

**Table 1:** Distribution of experimental sample data.

Types	Samples	Training Set	Testing Set
AF	1841	1472	369
AT	500	400	100
N	4800	3840	960
PAC	328	262	66
PVC	2106	1684	422
SBR	1855	1484	371
VT	294	235	59
Total	11,724	9377	2347

**Table 2:** Comparison of test results of different network models.

Network	Accuracy
Resnet50-ReLu	95.4%
Improved Resnet50-ReLu	97.5%
Improved Resnet50-SELU	98.3%

**Table 3:** Confusion matrix classified by proposed algorithm.

Real Category	Categories of Prediction							
	AF	AT	N	PAC	PVC	SBR	VT	Total
AF	357	0	4	1	3	2	2	369
AT	0	100	0	0	0	0	0	100
N	3	0	950	3	4	0	0	960
PAC	1	0	1	62	0	2	0	66
PVC	1	0	3	1	415	2	0	422
SBR	4	0	0	0	0	366	1	371
VT	2	1	1	0	0	0	55	59
Total	368	101	959	67	422	372	58	2347

**Table 4:** Performance metrics for each category.

Categories	Ppr	Sen	Spe	F1 Score
AF	96.7%	97.0%	99.3%	96.8%
AT	99.0%	100.0%	100.0%	99.5%
N	99.1%	98.6%	99.5%	98.8%
PAC	95.4%	95.4%	99.9%	95.4%
PVC	98.6%	98.6%	99.6%	98.6%
SBR	98.7%	98.9%	99.7%	98.8%
VT	94.8%	95.0%	99.9%	94.9%
Average	98.1%	97.6%	99.7%	97.6%

**Table 5:** Diagnosis results.

Patient ID	Age	Height	Weight	Past Medical History	Test Results
a	68	170 cm	90 kg	AF	N, AF
b	45	170 cm	85 kg	Incidental PAC	N
c	40	168 cm	70 kg	PAC	N, PAC
d	43	167 cm	85 kg	PVC, AF	N, AF

**Table 6:** Comparison with other methods.

Methods	Numbers	ACC	Ppr	Sen	Spe	F1
SNN [12]	4	97.9%	97.3%	80.2%	99.8%	88.0%
MLP [13]	4	94.8%	75.8%	78.9%	96.8%	77.3%
Wavelet-MLP [14]	5	97.6%	87.4%	83.4%	98.1%	85.4%
CNN-LSTM [15]	2	95.4%	95.2%	98.2%	86.5%	96.8%
Proposed	7	98.3%	97.1%	97.2%	99.7%	97.1%

## Data Availability

The data are not publicly available. The data presented in this study are available on request from the corresponding author.

## Abbreviations

The following abbreviations are used in this manuscript:

ECG	Electrocardiogram
ResNet	Residual network
SELU	Scaled exponential linear units
IoT	Internet of Things
LSTM	Long and short-term memory
CNN	Convolution neural network
ADC	Analog-to-digital conversion
BLE	Bluetooth Low Energy
GAFs	Gramian angular fields
2D	Two-dimensional
BN	Batch normalization
LTAF	Long-term AF
GASF	Gramian Summation Angular Field
PAC	Premature atrial contraction
PVC	Premature ventricular contraction
N	Normal
AF	Atrial fibrillation
VT	Ventricular tachycardia
SBR	Sinus bradycardia
AT	Atrial tachycardia
Ppr	Precision rate
Sen	Sensitivity
Spe	Specificity
Acc	Accuracy
MLP	Multi-layer perception
